# Copper that cancer with lysosomal love!

**DOI:** 10.18632/aging.100910

**Published:** 2016-02-25

**Authors:** Zaklina Kovacevic, Sumit Sahni, Des R. Richardson

**Affiliations:** Molecular Pharmacology and Pathology Program, Department of Pathology and Bosch Institute, The University of Sydney, New South Wales, 2006, Australia

**Keywords:** Dp44mT, DpC, P-glycoprotein, drug resistance, cancer

There is now extensive evidence that chelation of copper and/or iron can inhibit tumour growth in cell culture, animal models and human clinical trials [1]. These effects are due to the essential role these metal ions play in a variety of biological processes, including DNA synthesis. As such, there is growing interest in the development of new agents that surpass the anti-tumor activity of traditional metal-binding drugs such as desferrioxamine (DFO) [1].

Since entering this field over 20 years ago, our laboratory has been particularly interested in novel aroylhydrazones [2], and more recently thiosemicarbazones [3, 4], that bind iron and copper and inhibit tumor growth *in vitro* and *in vivo*. Our team have pioneered the development of a new class of agents, known as the di-2-pyridylketone thiosemicarbazones (DpT analogues) that show marked and selective anti-tumour activity [3, 4].

In a recent article, published in *Oncotarget* [5] we describe the favourable pharmacokinetic properties of these agents that will facilitate their entrance in clinical trials which will occur in 2016. However, the “road” to this point in the development of these potential anti-cancer drugs has been long and hard, with the adventure yet to end. Below we describe some of the exciting biochemical properties of these agents, in particular their ability to target the lysosome (lysosomotropism; “lysosome loving”) [6, 7]. This property is important in terms of the efficacy of these compounds to induce potent cytotoxic activity and overcome drug resistance, which is a major killer in advanced cancer [6].

The commercialization and clinical trials of these agents has been facilitated by extensive studies demonstrating the broad and potent activity of our first lead agent, di-2-pyridylketone 4,4-dimethyl-3-thiosemicarbazone (Dp44mT), in a variety of tumor cell-types *in vitro* and in *vivo* [3,4]. In fact, Dp44mT was more effective than much higher doses of the clinically-trialled thiosemicarbazone, Triapine^®^. Further, Dp44mT showed far less toxicity than Triapine^®^ [4].

While Dp44mT showed selective anti-tumour activity, it caused cardiac fibrosis in mice at high, non-optimal doses [4]. This led to a second generation of DpT analogues which showed high tolerability, potent anti-cancer activity and no cardiotoxicity [7]. Of these ligands, di-2-pyridylketone 4-cyclohexyl-4-methyl-3-thiosemicarbazone (DpC) was identified as the lead agent and possessed many advantages over Dp44mT, including: (1) greater anti-tumor activity than Dp44mT *in vivo* against tumor xenografts; (2) markedly improved tolerability when administered orally, as well as intravenously compared to Dp44mT that is toxic when given orally; (3) unlike Dp44mT, DpC does not induce oxidation of oxyhemoglobin to methemoglobin in erythrocytes; (4) DpC demonstrates a markedly greater plasma half-life in the rat, namely 10.7 h *versus* 1.7 h for Dp44mT [5]; (5) the anti-tumor efficacy of DpC exceeds that of gemcitabine (the “gold standard” chemotherapy for pancreatic cancer) *in vivo* against pancreatic xenografts; and (6) DpC does not induce cardiac fibrosis in nude mice even when given at markedly higher doses than Dp44mT [7]. Significantly, due to these favorable properties, DpC was commercialized and is expected to enter clinical trials in 2016 for the treatment of advanced and resistant cancers [7].

**Figure 1:**
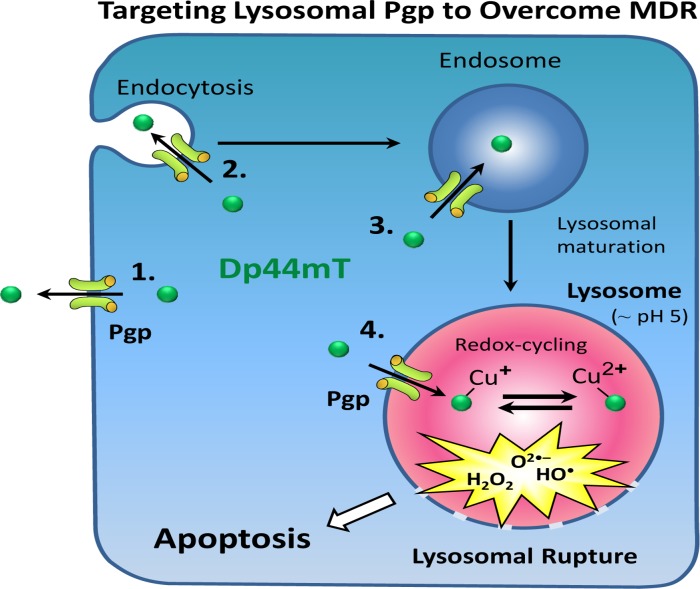
The ability of Dp44mT to overcome drug resistance is mediated by its ability to be transported into the lysosome by the drug transporter, P-glycoprotein (Pgp). Subsequently, within the lysosome, Dp44mT binds Cu and then redox cycles to generate cytotoxic reactive oxygen species (ROS) that subsequently results in lysosomal membrane rupture. The rupture of the lysosome leads to the release of enzymes such as cathepsins that trigger apoptosis and cell death [6, 7]. Hence, resistant cells with high Pgp expression then become sensitive to Dp44mT, leading to increased cell death, and thus, overcoming the multi-drug resistance (MDR) phenotype.

In terms of the detailed mechanism of action of these novel compounds, our studies examining the DpT analogues (*e.g*., Dp44mT) demonstrated that these agents target lysosomes due to their unique ionisation properties that causes them to be positively charged and trapped in the acidic lysosome (Fig. 1) [6,7]. This property results in lysosomal accumulation of Dp44mT, where high intracellular Cu levels reside. This leads to a redox-active Cu[Dp44mT] complex that generates reactive oxygen species (ROS) resulting in lysosomal rupture and tumor cell death [6, 7] (Fig. 1).

Significantly, we discovered that the drug transporter, P-glycoprotein (Pgp) that commonly mediates multi-drug resistance (MDR), is not only expressed and functional in the plasma membrane, but is also found intracellularly in lysosomes [6]. These data revealed Pgp-mediated sequestration of chemotherapeutics into lysosomes leads to MDR that could be exploited. This was verified when Pgp in the lysosomal membrane was shown to enhance transport of Dp44mT into this target organelle [6, 7] (Fig. 1). Such activity resulted in a marked increase in lysosomal rupture and cytotoxicity toward Pgp-expressing tumour cells in culture and tumors *in vivo* [6]. Similar results in terms of lysosomal targeting were also demonstrated for DpC. Thus, we discovered an innovative new strategy of using Pgp against itself to overcome the resistance it mediates in tumor cells [6, 7].

In summary, we have identified a new class of anti-tumor agents that have the novel property of targeting Cu in the lysosome that results in lysosomal rupture and cell death. This property is enhanced in cells that express high levels of the multi-drug resistance transporter, Pgp, leading to lysosomal accumulation and rupture, with the subsequent death of resistant tumor cells [6]. Further studies are now essential in order to target the lysosomal compartment more effectively using specially designed agents.
